# Isolated Resection of Segment I (Caudate Lobe): Is it Justified?

**DOI:** 10.1155/1996/87129

**Published:** 1996

**Authors:** Leslie H. Blumgart

**Affiliations:** Memorial Sloan-Kettering Cancer Center 1275 York Avenue New York New York 10021 USA

## Abstract

*Background*: Isolated caudate lobectomy is a challenging surgical procedure for which safe and reliable techniques have yet to be developed.

*Methods*: Isolated caudate lobectomy was performed by initial inflow control of the caudate lobe, full mobilization of the liver from the inferior vena cava by dividing all short hepatic veins, and parenchymal division dorsal to the major hepatic veins with a clockwise rotation of the liver while the liver was selectively devascularised by Pringle's maneuver and occlusion of the confluence of the major hepatic veins flush with the inferior vena cava.

*Results*: Two patients with cirrhosis underwent this procedure successfully without intraoperative hemodynamic instability or postoperative liver dysfunction.

*Conclusions*: This technique allows safe and truly selective excision of the caudate lobe without the need for occlusion of the inferior vena cava or venovenous bypass.


*HPB Surgery*, 1996, Vol.10, pp. 121–128
Reprints available directly from the publisher
Photocopying permitted by license only

© 1996 OPA (Overseas Publishers Association)
Amsterdam B.V. Published in The Netherlands
by Harwood Academic Publishers
Printed in Malaysia

### HPB INTERNATIONAL

**EDITORIAL & ABSTRACTING SERVICE**

JOHN TERBLANCHE, EDITOR
Department of Surgery,
Medical School · Observatory 7925
Cape Town · South Africa
Telephone: 27-21-406-6204
Telefax 27-21-448-6461
E-mail: JTER BLAN@UCT GSHI.UCT.AC.2A

### ISOLATED RESECTION OF SEGMENT I (CAUDATE LOBE): IS IT JUSTIFIED?

### ABSTRACT

*Yanaga, K., Matsumata, T., Hayashi, H., Shimada, M., Urata, K. and Sugimachi, K. (1994) Isolated hepatic caudate lobectomy. Surgery; 115:757–761.*

***Background:*** **Isolated caudate lobectomy is a challenging surgical procedure for which safe and reliable techniques have yet to be developed.**
***Methods:*** **Isolated caudate lobectomy was performed by initial inflow control of the caudate lobe, full mobilization of the liver from the inferior vena cava by dividing all short hepatic veins, and parenchymal division dorsal to the major hepatic veins with a clockwise rotation of the liver while the liver was selectively devascularised by Pringle's maneuver and occlusion of the confluence of the major hepatic veins flush with the inferior vena cava.**
***Results:*** **Two patients with cirrhosis underwent this procedure successfully without intraoperative hemodynamic instability or postoperative liver dysfunction.**
***Conclusions:*** **This technique allows safe and truly selective excision of the caudate lobe without the need for occlusion of the inferior vena cava or venovenous bypass. (Surgery 1994;115:757–761)**

**From the Department of Surgery II, Faculty of Medicine, Kyushu University, Fukuoka, Japan.**

KEYWORDS: Liver resection caudate lobe resection carcinoma liver

### PAPER DISCUSSION

This short technical presentation is of some considerable importance and draws attention to one of the very difficult problems in liver resection. The paper is significant in that it describes in a clear manner the anatomical and technical difficulties of isolated caudate lobe resection and emphasizes that the procedure can be carried out without total vascular isolation.

In writing this commentary I am pleased to concur with nearly all the points made by the authors, but there are some variations in the description of the anatomy and in the technique which I think are worth discussion.

In describing the anatomy of the caudate lobe the authors refer to the work of Kumon[1] and describe the caudate lobe as being in three parts. From this author's point of view the characterization of the caudate lobe of the liver into a left and right portion as described by Couinaud[2] is perhaps more appropriate since it emphasizes that some of the caudate lobe lies not only anterior but just to the right of the vena cava in an intraparenchymal position. It is this portion of the caudate, variable in size, which abuts the posterior surface of Segment IV, the limit being an oblique plane slanting from the left portal vein to the left hepatic vein and closely adjacent to the roots of the major hepatic veins as emphasized by the authors.

Another important anatomical point perhaps not sufficiently emphasized, although illustrated in one of the figures, is the fact that there is frequently a fairly dense band of fibrous tissue running from the left posterolateral caudate lobe behind the vena cava to join with Segment VII on the right side. Indeed, sometimes this band of tissue is replaced by hepatic parenchyma and it is this circumstance that the freeing of the caudate lobe can be extremely difficult and may involve sectioning of some parenchymal tissue behind the vena cava in order to afford mobilization.

The authors mention that, in the hepatic hilus, they perform cholecystectomy as the first procedure. We do not necessarily do this and I am not sure I understand why it is necessary to carry out cholecystectomy in order to remove the caudate lobe. Indeed, I am somewhat surprised that the authors are adding this to the procedure in hepatic resection *in the cirrhotic liver*, since it is well established that cholecystectomy of it's own, in patients with cirrhosis and portal hypertension, carries substantial risk.

I am in full accord with the authors' opinion that complete vascular isolation is not necessary for removal of the caudate lobe and, indeed,it can be difficult to achieve if there is a substantial caudate tumor present. I also agree that it is of value to occlude the roots of the major hepatic veins with a vascular clamp although it does seem from the authors' description as if they occlude all three major hepatic veins. I see no reason for this and it is our practise to occlude simply the left and middle hepatic veins, which usually have a common root, and not to interfere with venous drainage through the right hepatic vein in the performance of caudate lobectomy.

It may be perhaps of value to describe some of the key aspects of our own technique in caudate lobe resection, which while not different in essence, are indeed different in detail.

The major problems in caudate lobe dissection are in dissection and control of the retrohepatic caudate veins and anteriorly, in the presence of substantial tumors, in controlling bleeding from the middle hepatic vein. Resection of the caudate involves three essential steps:

1) Control of the inflow caudate blood supply from the left portal vein and left hepatic artery at the base of the umbilical fissure.
2) Dissection of the retrohepatic veins.
3) Severance of the liver parenchyma between the base of Segment IV and the left side of Segment VII to allow removal of the lobe. It is our practice that during isolation of the caudate blood supply and bile ducts the first and essential step is to lower the hilar plate. This separates the left portal vein and the left portal triad from the anterior surface of the caudate process and assists in full mobilisation of the caudate lobe. The left lobe of the liver is mobilized and turned to the right, and it is our practice to divide the ligamentous attachments on the left and mobilize the tip of the caudate lobe and it's left lateral margin from the inferior vena cava working through the lesser sac of the peritoneum prior to severance of the caudate veins. If there is difficulty during this dissection and it is evident that the caudate is embracing the vena cava posteriorly, no further attempt is made to free the lobe on the left but an approach is made from the right. Indeed, dissection of the retrocaudate veins from the vena cava can be done by a combination of dissection from the right and the left side so as to free the cava completely from the undersurface of the caudate lobe as high as the major hepatic veins. As emphasized by the authors it is important in isolated resection of the caudate lobe and, indeed, in resection of the caudate lobe in combination with extended right hepatic lobectomy to recognize the danger that may arise in hemorrhage from the middle hepatic vein should this be torn posteriorly[3]. In this instance we have found it valuable to isolate the left and middle hepatic veins by opening up to the tunnel between the left hepatic vein and the inferior vena cava at the upper end of the caudate. This is easily accomplished after division of the uppermost part of the ligamentum venosum. A clamp is then passed across the left and middle hepatic veins for temporary control during the parenchymal phase of the dissection of the portion of the caudate lobe lying anterior to the inferior vena cava. Finally, it is important to mention that some authors, in the presence of a large caudate lesion, have split the liver anteriorly in the Glissonian plane separating the right and left liver and dividing the liver along the right margin of Segment IV so as to approach the caudate intra-parenchymally from above. A combination of this technique with the methods describe above may be useful in such large lesions[4]. It is perhaps a minor criticism of this contribution that the authors do not mention this later contribution from Japan.

We have now resected a total of 21 complete caudate resections, 4 of whom underwent isolated caudate lobectomy[5]. The most common diagnosis was metastatic colorectal cancer in nine patients and the most common procedure was an extended left hepatic lobectomy with en bloc caudate lobectomy. The median operative time was five hours and the median blood loss was1,160 ml. Only one patient required intensive care and went on to die of liver failure. In none of our cases was vascular isolation employed but an intermittent Pringle maneuver combined with temporary occlusion of the left and middle hepatic veins was the method of choice[5].

In short, although only two cases of isolated hepatic lobectomy are reported this paper is of importance since there are but few reports of isolated caudate lobe resection, the most recorded by an author being the three cases reported by Colonna *et al.*[6] and the four cases referred to and submitted for publication by our own group.

There is no doubt that the development of hepatic resectional techniques and the understanding of the anatomy of the caudate lobe has resulted in an ability to resect the isolated caudate lobe safely, and that the addition of caudate lobectomy to major liver resection does not add significantly to the morbidity or mortality of the procedure.

### REFERENCES

1. Kumon M. (1985) Anatomy of the caudate lobe with special reference to portal vein and bile duct (in Japanese). Acta Hepatol Jpn 26: 1193–9.
2. Couinaud C. Couinaud C.,ed. (1989) Surgery anatomy of the liver revisited.Paris:Maugein& Ci 123–34.
3. Lerut J, Gruwez JA, Blumgart LH.(1990) Resection of the caudate lobe of the liver. Surg Gynecol Obstet 171: 160–2.
4. Yamamoto J, Takayama T, Kosuge T, et al. (1992) An isolated caudate lobectomy by the transhepatic approach for hepatocellular carcinoma in cirrhotic liver. Surgery 111: 699–702.
5. Bartlett D, Fong Y, Blumart LH. (1995) Complete resection of the caudate lobe of the liver-technique and results. *British Journal of Surgery,* In Press.
6. Colonna JO, Shaked A, Gelabert HA, Busuttil RW. (1993) Resection of the caudate lobe through "bloody gultch". Surg Gynecol Obstet 176: 401–2.

Leslie H Blumgart, MD, FACS, FRCS
Chief:Hepatobiliary Service
Memorial Sloan-Kettering Cancer Center
1275 York Avenue
New York, New York 10021
United States of America

### LIVER RESECTION: PROLONGED INFLOW OCCLUSION IN HUMAN CIRRHOTIC LIVERS

### ABSTRACT

*Kim, Y.I., Nakashima, K., Tada, I., Kawano, K. and Kobayashi, M. (1993) Prolonged Ormothermic Ischaemia of Human Cirrhotic Liver during Hepatectomy: A Preliminary Report. Br J Surg, **80**: 1566–1570.*

**To evaluate the tolerance of the cirrhotic liver to extended warm ischaemia, 47 patients with cirrhosis who underwent liver resection over a 4-year period were studied retrospectively. Three groups of patients were identified. In group 1 (14 patients) liver resection was performed under conditions of portal triad occlusion ranging from 50 to 75 (mean 57.1) min. Group 2 (12 patients) was treated with portal occlusion for a period ranging from 30 to 42 (mean 33.1) min. Group 3 comprised 21 patients who underwent hepatectomy using conventional techniques. Mean blood loss was significantly**

